# Exploring sustainable livelihood options for COVID-impacted rural communities in Bangladesh

**DOI:** 10.1016/j.heliyon.2024.e38664

**Published:** 2024-09-27

**Authors:** Abul Bashar, Neaz A. Hasan, Mohammad Mahfujul Haque

**Affiliations:** a Department of Aquaculture, Bangladesh Agricultural University, Mymensingh, 2202, Bangladesh; b Department of Plant and Environmental Science, University of Copenhagen, Thorvaldsensvej 40, 1871, Frederiksberg C, Denmark; c Department of Fisheries and Marine Bioscience, Bangabandhu Sheikh Mujibur Rahman Science and Technology University, Gopalganj, 8100, Bangladesh

**Keywords:** COVID, Sustainable livelihood, SLA framework, Policy intervention

## Abstract

The COVID pandemic paralyzed all economy-generating sectors of Bangladesh, putting abrupt pressure on livelihood-making. In this study, two sequential surveys were conducted in the Mymensingh district of Bangladesh to gain a deep understanding of the impacts of COVID on job status and post-COVID job preference. The effectiveness of preferred occupations in livelihood resilience within the Sustainable Livelihood Approach (SLA) framework was also empirically analyzed and people-driven policies were recommended for sustaining livelihoods. About 35.96 % of respondents experienced short-to long-term unemployment and migrated to rural areas. The groups most severely affected were predominantly engaged in both formal and informal employment, characterized by lower levels of education, older age brackets, and predominantly female (45 out of 114). In the COVID situation, a large portion of respondents adopted agriculture (18.42 %), aquaculture (10.53 %), livestock farming (5.26 %), and small businesses (13.27 %) as their means of livelihood. However, for post-COVID preferences, respondents remained limited to only 13 job categories, considering aquaculture (22.02 %), agriculture (19.90 %), and livestock farming (26.22 %) as their most desired sectors. Considering all five capitals within the SLA framework, aquaculture and livestock farming were found to be more effective in bringing livelihood resilience within COVID-affected rural communities than agriculture. However, the greater advantages that aquaculture offers in achieving post-COVID livelihood led the authors to consider aquaculture as a decisive choice for policy recommendation. Therefore, the study proposed 28 people-oriented policies under four broad categories (financial support, technical assistance, marketing and logistics, and governance and institutional) in making sustainable livelihoods from aquaculture where financial support takes precedence as their top priority.

## 1 Introduction

Since the outbreak of COVID, the economy-generating sectors in almost all countries and territories around the world have been impacted [1,2], edging towards a long and challenging economic recession [3]. The shock from COVID has devastated the world economy, even in the most economically resilient countries, causing global economic growth to decline by 4.9 % since the second half of 2020 [4]. Heading to the new normal, measures initiated by most countries to elevate GDP have been considered the most robust reversion in the last 80 years against any economic tremor [5]. However, according to the World Bank report, global GDP remains 3.2 % below the projections made in the pre-COVID era [6]. The World Trade Organization also warned that world merchandise and market exchange would fall in late 2022 and remain subdued in 2023, with a 1 % growth reduction from an estimated 3.2 %, due to the simultaneous effects of the COVID-induced economic recession and the Ukraine-Russia war [7].

Historical evidence showed that previous pandemics, such as the Spanish flu, SARS, Swine flu, Avian flu, Ebola, and Zika virus, flexed economic activities and aggravated unemployment to a significant extent [8,9]. However, the outbreak of COVID has caused the worst economic depression ever, resulting in extreme uncertainty in livelihood [10,11], a steep drop in income, and a downtick in consumption [12,13]. It was expected that globally about 3.3 billion people would be at risk of losing their livelihood due to workforce vulnerability [14]. China exceptionally geared up its GDP with a solid rate of 8.5 % in 2021 [5], however, impacts from the pandemic are projected to continue its legacy for a longer time in Low- and Middle-income Countries (LMICs). This is due to the fragile economy, a sharp decline in investment, financial inflation, skill erosion, and the least recruitment against the highest job cut in LMICs. The situation is more detrimental to Bangladesh compared to other developing countries due to a significant portion of its population enduring sub-standard living conditions below the extreme poverty line [15]. This predicament is exacerbated by the nation's heavy reliance on export and import for daily needs [16], pervasive knowledge disparities [17], and a lack of robust national preparedness and post-pandemic support system to safeguard vulnerable communities [18].

Strict regulations on human movement and transportation systems have effectively curtailed the spread of the COVID pandemic in Bangladesh but have led to a sharp increase in economic debts and a synchronized post-pandemic recession, leaving very few opportunities for recovery [19]. The pandemic and consequential austerity measures have profoundly impacted the economy of various sectors (aviation, travel, tourism, remittances, manufacturing, and agricultural production), eliciting significant concerns regarding people's livelihood. According to a report by the Bangladesh Institute of Development Studies, the pandemic has caused unemployment for about 13 % of people [20], increasing the national unemployment rate to 5.30 % from around 4 % in 2019 and herding an additional 7 million people to the unemployed figure [21]. More than 80 % of laborers engaged in informal jobs in urban areas have reported financial losses [22]. Amidst unemployment or low-paid employment, millions of urban working-class people migrated to rural areas [23]. This migration put additional pressure on rural communities, leading to a serious crisis with an unreciprocated question of livelihood outcome due to limited resources and opportunities existing in remote areas. The far-reaching consequences of these livelihood losses are likely to exacerbate poverty, food insecurity, health crises, gender discrimination, and socioeconomic inequalities [24,25]. Despite substantial efforts by the central government, the pandemic has pushed an additional 2 % of the population into extreme poverty [26], prompting concerns about the progress in combating poverty and fostering sustainable livelihoods.

Livelihood is considered a synergistic target that directly influences both economic and social dimensions, while also offering some co-benefits for the environment. As a consequence, ensuring sustainable livelihood is contemplated as a legitimate and deliberate contribution to chasing the national development agenda of LMICs [27]. Assessing the impact of job market disruptions during the shockwaves of the COVID era and evaluating post-COVID opportunities are crucial for understanding and addressing long-term challenges that may persist for decades. This understanding is vital for strategically planning the recovery and advancement of economies in the post-pandemic period. While recent studies have examined potential livelihood options and economic activities for various communities (following descriptive and theoretical modeling) [[28], [29], [30]]there remains a gap in exploring post-COVID livelihood options for rural communities, particularly integrating SLA in any LMICs, including Bangladesh. Therefore, in this study, we conducted two surveys to identify potential livelihood strategies for COVID-affected rural communities of Bangladesh that are expected to offer co-benefits across the longer-term resilience. Additionally, we present individual-aware recommendations from community people to help policymakers to formalize community-oriented policies.

## 2 Materials and method

### 2.1 Study approach and analytical framework

A case study using a triangulation approach was employed, focusing on gaining deep insight into the impacts of COVID and restoring livelihood resilience through quantitative evaluation of different occupations within the SLA framework. The notion of “triangulation” involved i) collecting social data regarding the impacts of COVID on the livelihoods of rural communities and their attempts to recover; ii) conducting a quantitative assessment of livelihood resilience within the SLA framework; and finally, iii) receiving policy recommendations from community members to guide a policy brief. Before conducting surveys, we sought consent and ethical approval (BAURES-2021) from the Bangladesh Agricultural University Research System (BAURES) due to the involvement of human participants in the study design.

Among the available tools, the Sustainable Livelihood Framework (SLF) is the most significant for identifying potential challenges associated with livelihood vulnerability and establishing an appropriate nexus between solution-making and institutional support [31,32]. Given the nature of the COVID pandemic and the associated livelihood disruption through the destruction of productive assets, many scientists have proposed SLF in defining and recovering COVID-induced livelihood vulnerabilities [28,29]. However, none of these studies have adopted a numerical approach to index livelihood components needed to design COVID-affected livelihoods. To circumvent the theoretical blind spot in scientific prediction, recent literature has argued for empirical illustrations of SLF through indexing capital indicators to improve understanding of how and to what extent different capitals allow people to use coping mechanisms and for advocating potential interventions [33]. Therefore, rather than considering a simple top-down checklist, we instigated SLF to analyze the livelihood resilience index to ensure maximum access to existing resources.

### 2.2 Context of the study area

**Fig. 1:**
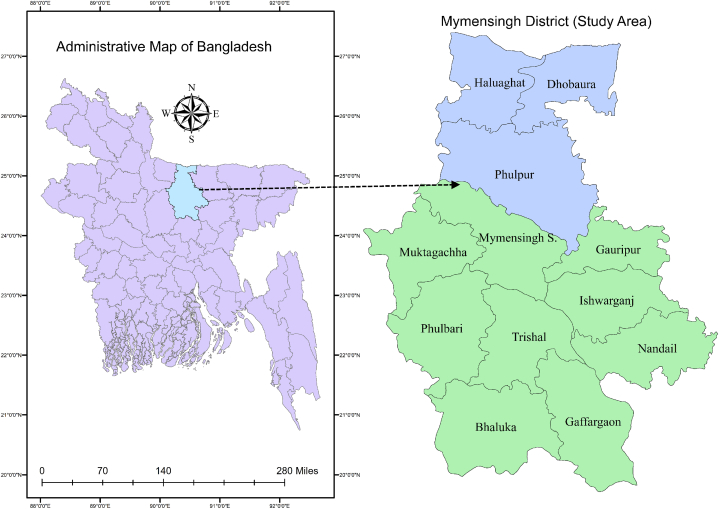
Map of the study area. Subdistricts of Mymensingh indicate the locations of the preliminary survey conducted among which blue subdistricts represent the area covered during the key-informant interview (the administrative boundary layer of the map was extracted from the shapefile created by DivaGIS). (For interpretation of the references to colour in this figure legend, the reader is referred to the Web version of this article.)

The study focused on the Mymensingh district of Bangladesh (Fig. 1) as an exemplary setting, which typically resembles the COVID-affected rural context throughout the country. The extraordinary climate and biophysical properties of Mymensingh exhibit the potential for compensating for livelihood losses in post-COVID situations. The presence of fertile croplands on the banks of the Old Brahmaputra and seasonal rainfall offer marginal farmers the opportunity to cultivate a variety of crops and vegetables, with greater potential to earn livelihoods from fishing and other ecosystem benefits.

### 2.3 Data collection

#### 2.3.1 Preliminary survey and selection of sub-districts

To select three specific sub-districts for empirical data collection, a cross-sectional survey was conducted in all 12 sub-districts of Mymensingh from January to June 2021. A total of 228 respondents (at least 15 individuals from each sub-district), engaged in any pre-COVID job, were physically surveyed maintaining proper health precautions, or surveyed through telephone. To achieve the research goals, section A of the questionnaire aimed to gather information on demographic attributes such as age, gender, education level, household size, employment type, etc., while section B was related to the occupational and economic status of respondents (see supplementary file 1). To understand the impact of COVID on livelihood, the difference between pre- and in-COVID situations was shown for the number of people involved and their average monthly income. Based on the employment stratification from the preliminary survey, three sub-districts (Haluaghat, Dhobaura, and Phulpur) of Mymensingh were identified as having the worst wave from COVID and the fewest opportunities to recover their livelihoods in the post-COVID situation for further exploration.

#### 2.3.2 Key-informant interview

Based on the preliminary survey findings and using the activity choice approach [34]; agriculture, aquaculture, and livestock farming were identified as the three most common job options. A key-informant interview was undertaken to assess livelihood capital indices, aiming to recognize the most viable job opportunities that may help them recover from the COVID crisis (see Supplementary file 2 for the questionnaire). A total of 30 participants were approached in each sub-district, however, with complete consent, 25, 22, and 24 participants were interviewed in Haluaghat, Dhobaura, and Phulpur, respectively. To make the KII more directive (limited to only selected occupations) and purposive, participants involved in agriculture, aquaculture, and livestock farming were selected randomly and who have at least a higher secondary degree (to gain better insights). Individual values on a 5-point scale (1 for very poor, 2 for poor, 3 for moderate, 4 for strong, and 5 for very strong) were collected to reflect the degree of support that an option could provide in bringing livelihood resilience.

Lastly, 47 respondents who had an association with the aquaculture business in post-COVID or in-COVID situations and/or intended to adopt aquaculture as their desired profession were approached to rank recommendations (identified by authors from literature and personal experience) needed to improve the feasibility of the aquaculture business. Data were collected through a simple divergent interview (see Supplementary file 3 for the questionnaire), and respondents were asked to assign relative importance to each recommendation using a simple Likert scale from 1 to 5, where 5 indicated the most desired recommendation.

### 2.4 Computation and data management

The resilience indices of capital components were determined through the following equation (Eq. (1)):

[1] $$R_{ic}=W_{ic}\times I_{ic}$$

Where, *R*_*ic*_*, W*_*ic*_, and *I*_*ic*_ indicate the resilience value of the *i*th component, individual weight for the *i*th component, and average Indicator value given by respondents for the *i*th component, respectively.

All resilience values (*R*_*ic*_ ….*R*_*nc*_) were summed up to give rise overall resilience value for each capital and the resilience index of each livelihood option was measured through the following equations (Eq. (2) and Eq. (3)):

[2] $$R_{ia}=R_{ic}+R_{jc}+R_{kc}+\ldots \ldots \ldots +R_{nc}$$

[3] $$R_{ilo}=R_{p}+R_{n}+R_{h}+R_{s}+R_{f}$$

R_*ilo*_ indicates the resilience index for *i*th livelihood options, calculated as the whole of all capital's overall values (*R*_*p*_ = overall resilience value for physical capital; *R*_*n*_ = overall resilience value for natural capital; *R*_*h*_ = overall resilience value for human capital; *R*_*s*_ = overall resilience value for social capital; *R*_*f*_ = overall resilience value for economic capital).

For data management and simple qualitative analysis, we used Microsoft Excel (2010). To visualize sub-district-wise respective data of job loss, transition, and stability were symbolized in maps using ArcGIS software (version 10.8). Gephi software was used to present occupational transition and desires networks, considering the number of respondents as edge weight. The significance of overall resilience values and resilience index across the three livelihoods (aquaculture, agriculture, and livestock farming) was evaluated using the Tukey Post Hoc test at a 5 % level of significance in IBM SPSS Statistics (version 25).

## 3 Results and discussion

### 3.1 COVID hit and employment status

**Fig. 2:**
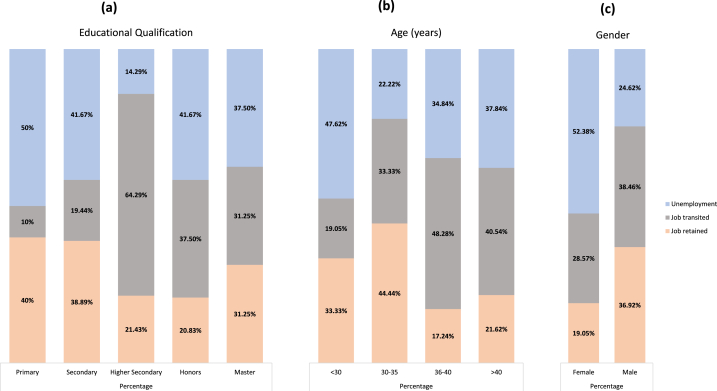
Demographics of respondents affected by COVID, showing the percentage of job retained, job transited and unemployed in the in-COVID situation regarding educational qualification (a), age (b), and gender (c).

During the COVID pandemic and resulting lockdowns, several challenges arose in the job market, including temporary or permanent job loss and reduced income. Seasonal and migrant workers, who were typically less paid and unprotected, were hit hard by the pandemic. People with lower educational qualifications, such as those who had only completed primary or secondary school, were found to be highly affected, with the highest levels of unemployment (50 % and 41.67 %, respectively) and the lowest opportunities (10 % and 19.44 %, respectively) to transition to new occupations. As the government mandated maintaining proper health care and strict vaccine security, workers with higher secondary education and substantial skills and experience were preferred over those with less educational attainment (primary to secondary level) (Fig. 2a). People aged less than 30 and over 40 were found to be at higher risk of unemployment (47.62 % and 37.84 %, respectively) (Fig. 2b). Unemployment was found to be positively correlated with gender: females (52.38 % unemployed) were significantly more affected than males (24.62 % unemployed) across the study (Fig. 2c).

**Fig. 3:**
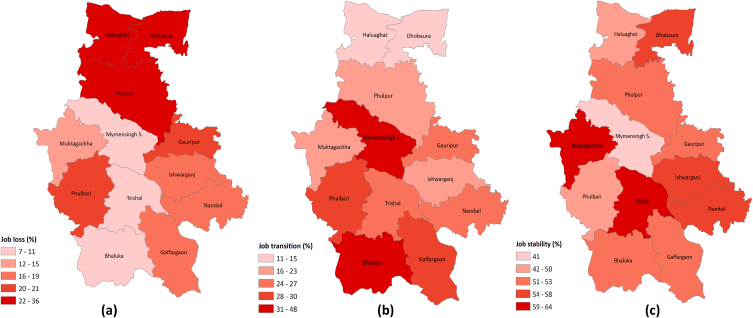
Employment stratification of rural people in Mymensingh shaped by COVID posed lockdown: (a) % of people lost their employment during the lockdown and remain unemployed; (b) % of people changed their workforce during lockdown; (c) % of people still employed in their previous workforce in all subdistricts of Mymensingh.

Being geographically distant from the industrial hub, the sub-districts Dhobaura and Haluaghat were found to have a higher prevalence of unemployment (32 % and 37 %, respectively), whereas even under drastic restrictions, minimal unemployment was reported in industrial sub-districts such as Bhaluka (7 %) and Trishal (9 %) (Fig. 3a). The Mymensingh city exhibited the most significant job transition rate at 48 %, attributable to its diverse job market, especially catering to individuals with lower incomes. Conversely, Dhobaura is less developed and diversified in terms of livelihoods, and therefore showed the lowest level of job transition (11 %) (Fig. 3b). Although workers in Bhaluka and Mymensingh faced the lowest level of unemployment, the job switching rate was higher due to reduced payment in previous positions and/or a higher chance of moving towards COVID mitigation businesses, such as venturing with face masks and sanitizers [35]. With Bhaluka and Trishal, exceptionally, job stability in Dhobaura was found to be higher (Fig. 3c), where agricultural practice remains dominant over any other informal jobs.

### 3.2 In-COVID occupational transition and post-COVID livelihood preference

**Fig. 4:**
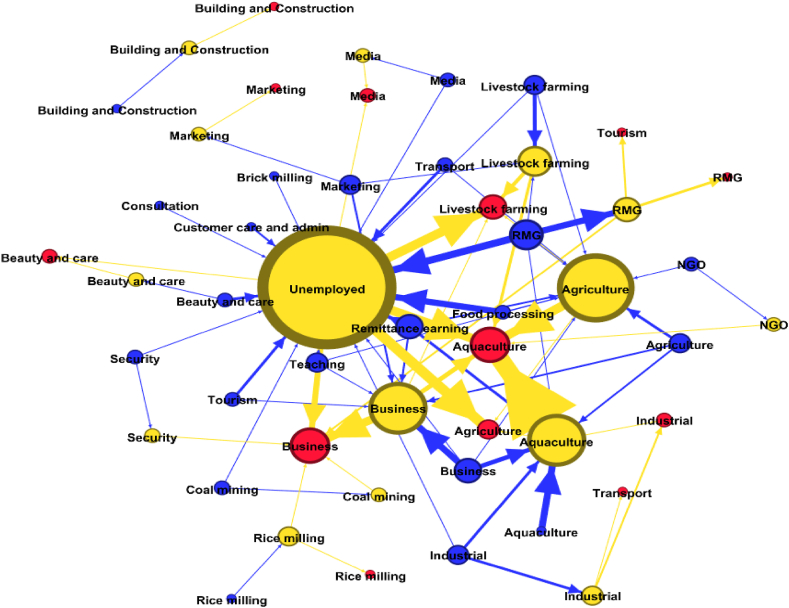
Occupational network showing pre-COVID (blue), in-COVID (yellow), and desired (red) job categories. Edge width and the degree of centrality reflect the number of times respondents changed their occupation, while node size is determined by the degree of transition. (For interpretation of the references to colour in this figure legend, the reader is referred to the Web version of this article.)

The data showed that COVID disproportionately affected all categories, with about 35.96 % (41 out of 114) of respondents experiencing short-to long-term unemployment and returning to rural areas (Table 1). Respondents engaged in food processing industries in pre-COVID were found to be the highest unemployed (87 %), followed by beauty and care (75 %), tourism and transportation (75 %), education (60 %), coal mining (50 %), media (50 %), security services (50 %), and the RMG sector (44.44 %), respectively (Fig. 4). While no shift was observed in aquaculture, beauty and care, and rice mills, no retainment was observed for consultation services. In the in-COVID situation, a large portion of respondents adopted agriculture (18.42 %), aquaculture (10.53 %), livestock farming (5.26 %), and small businesses (13.27 %) as their means of livelihood because of optimal support from natural resources and established settings.

**Table 1:** Difference in the number of people involved in and income from different occupations during pre-COVID and in-COVID situations.

Occupation	Number of people involved			Average Income		
	Pre-COVID	In-COVID	Difference (%)	Pre-COVID	In-COVID	Difference (%)
Agriculture	7	21	**↑** 200.00	3142.86	2653.85	**↓** 15.56
Aquaculture	7	12	**↑** 71.43	30714.29	19363.64	**↓** 36.96
Beauty and care	4	1	**↓** 75.00	28250.00	10000.00	**↓** 64.60
Brick mill	1	0	**↓**100.00	12000.00		
Building and Construction	1	1	0.00	18000.00	15000.00	**↓** 33.00 %
Business	14	15	**↑** 7.14	23714.29	18105.26	**↓** 23.65
Coal mining	2	1	**↓** 50.00	13500.00	9000.00	**↓** 33.33
Consultation	1	0	**↓** 100.00	65000.00	–	–
Customer care and admin	2	0	**↓** 100.00	29000.00	–	–
Educational institute	5	0	**↓** 100.00	24400.00	–	–
Farming	6	6	0	13333.33	10500.00	**↓** 21.25
Industrial	7	3	**↓** 57.14	37142.86	37333.33	**↑** 0.51
Marketing	4	1	**↓** 75.00	35500.00	25000.00	**↓** 29.58
Media	2	1	**↓** 50.00	34000.00	32000.00	**↓** 5.88
NGO	2	1	**↓** 50.00	22500.00	25000.00	**↑** 11.11
Processing	8	0	**↓** 100.00	28625.00	–	–
Remittance worker	11	0	**↓** 100.00	65909.09	–	–
Rice mill	2	2	0	9000.00	7500.00	**↓** 16.67
RMG	18	7	**↓** 61.11	28857.14	18000.00	**↓** 37.62
Security	2	1	**↓** 50.00	15000.00	11000.00	**↓** 26.67
Tourism	4	0	**↓** 100.00	31750.00	–	**-**
Transport	4	0	**↓** 100.00	15500.00	–	**-**
Unemployed	0	41	–	–	–	–

Considering different matrices such as profit-making, environment, sustainability, and security, 42.47 % of respondents expressed their dissatisfaction with in-COVID jobs and were likely to change their occupations to restore professional satisfaction. Among all, 18.42 % of respondents who continued their pre-COVID jobs in the in-COVID situation were found to prefer their existing one, with aquaculture (33.33 %) and RMG (23.81 %) at the top tier. However, aquaculture (61.29 %) and agriculture (28.21 %) were the most attractive options for those who desired to move for a better job and for the unemployed force, respectively. Overall, preferences remained limited to only 13 job categories, with no choice for brick mill, coal mining, consultation, customer care and admin, teaching, NGO, food processing, and remittance earning (termed forced jobs - adopted for subsistence in the pandemic era). Among these 13 categories, aquaculture (22.02 %), agriculture (19.90 %), and livestock farming (26.22 %) were reported to be the most desired sectors for their next investment (Fig. 4). Therefore, these three professions were considered our decisive choices for further quantitative analysis to screen out the best option through the SLA framework.

Job satisfaction is essential for increasing the motivation and efficacy of an employee [36] which is strongly linked to mental health and social problems [37]. In rural settings, destitute people have adopted on-farm and other small-scale non-farm businesses during the COVID situation, as evidenced in a study carried out by Das et al. [38]. Although rural areas of Bangladesh were previously considered to lack access to institutional support and services, marketing infrastructures, and financial aid; recent advancements in electrification and power development have blessed rural areas with farming intensification [39]. Moreover, the richness of natural resources with ecosystem services has attracted people to on-farm jobs, which have produced many success stories [40]. Along with financial flow, these on-farm occupations ensure food security to a greater extent than non-farm jobs, which is the main argument behind the preference of people.

### 3.3 Role of preferred occupations in post-COVID livelihood resilience index

This subsection presents the estimated livelihood resilience supported by aquaculture, agriculture, and livestock sectors within the sustainable livelihood framework. A total of 32 capital components (7 physical, 9 natural, 6 financial, 4 human, and 6 social) were identified and confirmed through a literature review and the author's experience (Table 2). Individual weights were assigned to each component, and the results showed that physical capitals occupied the highest share (26.53 %), followed by natural (24.63 %), financial (22.31 %), social (17.97 %), and human (8.97 %) capitals. This suggests that infrastructural arrangements are the most important factor for successful livelihoods, where human capacity plays a very limited role. From an individual standpoint, respondents identified economic returns (4.98 %) and marketing aspects (4.97 %) as the most prioritized factors, while labor capability received minimum attention (1.02 %).

**Table 2:** Capital components needed to sustain rural livelihood with their given weights.

Capitals	Abbreviation	Components	Individual Weight (InW)^∗^
Physical	P1	Basic infrastructure for farming	0.0237
	P2	Transportation and logistics support	0.0411
	P3	Availability of machinery, farm equipment, and compliances	0.0389
	P4	Input supply and availability	0.0451
	P5	Market stability and access	0.0497
	P6	Electricity and power supply	0.0405
	P7	Value addition facilities	0.0263
Natural	N1	Availability of arable lands	0.0456
	N2	Soil quality	0.0203
	N3	Meteorological and climatic conditions	0.0189
	N4	Water availability and supply	0.0435
	N5	Flood control and storm protection	0.0311
	N6	Seasonality/timeframe	0.0215
	N7	Ecosystem services	0.0154
	N8	Disease history and management	0.0345
	N9	Resource management	0.0156
Financial	F1	Capital support (bank loan, easy access to credits)	0.0482
	F2	Economic return (BCR^b^)	0.0498
	F3	National subsidies	0.0305
	F4	Alternative income generation opportunity	0.0345
	F5	Savings	0.0387
	F6	Insurance	0.0214
Human	H1	Education, skill, and knowledge	0.0299
	H2	Physical compatibility	0.0102
	H3	Ability to work/labor availability	0.0299
	H4	Mental and physical health support	0.0156
Social	S1	Collaboration, networking, and cooperation	0.0256
	S2	Social security	0.0309
	S3	Gender issues	0.0373
	S4	Social hierarchy and evaluation	0.0355
	S5	Availability of knowledge and extension services	0.0158
	S6	Farmer's confederation	0.0345

^∗^Individual weight is calculated as the ratio between average individual value and total average value for indicators.

a Benefit cost ratio.

While Food and Agricultural Organization sets a “food-first” mandate and Das et al. [38] discourage centralized livelihood activities based on cities and industrial areas, we focused solely on rural on-farm jobs for our choice of occupation. Table 3 shows the resilience scores and benchmark rankings for three livelihood options (agriculture, aquaculture, and livestock farming) to determine which one is the most suitable based on available resources. Aquaculture and livestock farming were found to have higher associations with the overall index value of physical capital compared to agriculture. Livestock and aquaculture were the top-ranked options for supporting P1 and P5, respectively. Agriculture was believed to receive the highest support from N1, while N7, N8, and N9 were expected to favor mostly aquaculture. Aquaculture and livestock farming were better options for financial resilience, even though agriculture was found to receive higher financial support from the government. Additionally, aquaculture and livestock sectors were preferred more by the respondents because these sectors offer better potential for an integrated culture system, leading to better profit security with lower economic risks. Aquaculture was found to be the most suitable and employee-friendly job for rural jobless people, with the highest labor availability. However, livestock farming was reported to require a lower level of physical compatibility (F2). Both aquaculture and livestock farming were trusted to improve the physical and mental health of rural households and provide higher levels of macro- and micro-nutrients [[41], [42], [43], [44]]. Additionally, aquaculture was socially weighed higher and supposed to support gender issues more than agriculture and livestock, however, the agriculture sector was acknowledged more in knowledge production, dissemination, and technical service provisions.

**Table 3:** Calculated livelihood resilience indices of agriculture, aquaculture, and livestock within the sustainable livelihood framework.

Capitals	Components	W_*ic*_^∗^	Aquaculture		Agriculture		Livestock farming		*P* value
			I_*ic*_^∗∗^	R_*ic*_^∗∗∗^	I_*ic*_	R_*ic*_	I_*ic*_	R_*ic*_	
Physical	P1	0.0237	3.56	0.085 ± .005^a^	4.55	0.108 ± .010^b^	4.85	0.115 ± .008^b^	0.008
	P2	0.0411	4.12	0.169 ±0 .080	3.88	0.159 ± 0.280	4.62	0.190 ±0 .230	0.270
	P3	0.0389	3.98	0.1546 ±0 .017	4.78	0.1857 ±0 .026	4.36	0.1694 ±0 .026	0.329
	P4	0.0451	4.86	0.2192 ±0 .007	4.92	0.2219 ±0 .024	4.70	0.2119 ±0 .022	0.816
	P5	0.0497	4.46	0.2218 ± .012^b^	2.30	0.1144 ± .025^a^	2.80	0.1392 ± .010^a^	0.001
	P6	0.0405	3.98	0.1613 ±0 .024	3.40	0.1378 ±0 .020	4.60	0.1864 ±0 .045	0.243
	P7	0.0263	4.28	0.1126 ± .009^b^	1.80	0.0473 ± .008^a^	3.56	0.0936 ± .012^b^	0.001
	**⅀R**_***p***_ =			**1.124 ± 0.029**^**b**^		**0.974 ± .037**^**a**^		**1.105 ± .016**^**b**^	**0.001**
Natural	N1	0.0456	3.18	0.1449 ± .010^a^	4.64	0.2114 ± .027^b^	4.85	0.2209 ± .013^b^	0.004
	N2	0.0203	4.56	0.0926 ±0 .015	4.64	0.0942 ±0 .013	4.92	0.0999 ±0 .018	0.832
	N3	0.0189	3.78	0.0715 ±0 .005	3.22	0.0609 ±0 .013	3.56	0.0673 ±0 .013	0.484
	N4	0.0435	4.24	0.1846 ±0 .034	4.10	0.1785 ±0 .022	4.46	0.1942 ±0 .029	0.804
	N5	0.0311	4.35	0.1355 ±0 .014	2.98	0.0928 ±0 .027	4.34	0.1352 ±0 .017	0.068
	N6	0.0215	3.86	0.0830 ±0 .007	3.56	0.0766 ±0 .007	4.12	0.0886 ±0 .009	0.253
	N7	0.0154	3.02	0.0464 ± .003^b^	0.86	0.0132 ± .005^a^	0.44	0.0068 ± .003^a^	<0.001
	N8	0.0345	3.98	0.1371 ± .003^b^	4.46	0.1537 ± .006^b^	2.34	0.0806 ± .007^a^	<0.001
	N9	0.0156	4.04	0.0628 ± .004^b^	3.88	0.0603 ± .011^ab^	2.86	0.0445 ± .005^a^	0.037
	**⅀R**_***n***_ =			**0.9533 ± 0.028**		**0.9410 ± 0.031**		**0.9321 ± 0.029**	**0.690**
Financial	F1	0.0482	3.22	0.1553 ± .016^ab^	3.86	0.1861 ± .008^b^	3.10	0.1495 ± .017^a^	0.044
	F2	0.0498	3.56	0.1775 ± .007^b^	1.56	0.0778 ± .012^a^	3.78	0.1884 ± .017^b^	<0.001
	F3	0.0305	2.10	0.0640 ± .006^a^	4.06	0.1237 ± .029^b^	2.88	0.0877 ± .007^ab^	0.017
	F4	0.0345	4.22	0.1454 ± .005^b^	1.26	0.0434 ± .010^a^	4.48	0.1544 ± .024^b^	<0.001
	F5	0.0387	3.98	0.1539 ± .005^b^	2.04	0.0789 ± .012^a^	3.48	0.1346 ± .033^b^	0.010
	F6	0.0214	2.56	0.0548 ±0 .008	2.28	0.0488 ±0 .004	2.02	0.0432 ±0 .006	0.222
	**⅀R**_***f***_**=**			**0.7544 ± .015**^**b**^		**0.5563 ± .024**^**a**^		**0.7557 ± .035**^**b**^	**< 0.001**
Human	H1	0.0299	3.52	0.1052 ±0 .011	4.56	0.1363 ±0 .024	3.28	0.0980 ±0 .008	0.055
	H2	0.0102	3.72	0.0380 ± .004^ab^	2.88	0.0294 ± .001^a^	4.06	0.0415 ± .005^b^	0.022
	H3	0.0299	3.76	0.1125 ± .007^b^	2.22	0.0664 ± .006^a^	3.10	0.0928 ± .012^b^	0.002
	H4	0.0156	4.12	0.0642 ± .009^b^	2.86	0.0446 ± .005^a^	3.98	0.0620 ± .006^b^	0.021
	**⅀R**_***h***_ =			**0.3206 ± .011**^**b**^		**0.2759 ± .024**^**a**^		**0.2929 ± .020**^**ab**^	**0.046**
Social	S1	0.0256	4.26	0.1091 ±0 .022	4.88	0.1249 ±0 .015	4.36	0.1116 ±0 .014	0.522
	S2	0.0309	3.34	0.10336 ±0 .010	2.74	0.0848 ±0 .020	3.06	0.0947 ±0 .006	0.304
	S3	0.0373	4.22	0.1576 ± .031^b^	1.88	0.0702 ± .017^a^	3.04	0.1135 ± .011^ab^	0.008
	S4	0.0355	4.76	0.1690 ± .012^b^	3.28	0.1164 ± .005^a^	3.58	0.1271 ± .011^a^	0.001
	S5	0.0158	2.84	0.0450 ± .006^ab^	4.16	0.0659 ± .013^b^	2.62	0.0415 ± .004^a^	0.029
	S6	0.0345	3.56	0.1227 ± .017^b^	2.88	0.0992 ± .008^ab^	2.36	0.0813 ± .009^a^	0.014
	**⅀R**_***s***_ =			**0.7071 ± .022**^**b**^		**0.5613 ± .040**^**a**^		**0.5687 ± .018**^**a**^	**0.001**
	**⅀R**_***ilo***_**=**			**3.8589 ± 0.0279**^**b**^		**3.3129 ± 0.206**^**a**^		**3.6654 ± 0.393**^**b**^	**0.002**

^∗^Resilience value; ^∗∗^Individual weight; ^∗∗∗^Average Indicator value given by respondents

Over the past two decades, farmers have been motivated to convert their agricultural lands into aquaculture ponds due to better economic output [45]. Livestock farming has also emerged as a profitable business for rural communities due to more crop cycles practiced in a single year and huge market demands. However, poultry farming faces frequent disease outbreaks and higher treatment costs, posing risk to both production and investment [46]. While shrimp farming as the nexus of aquaculture also has a greater disease burden, most finfishes generally exhibit lower susceptibility to diseases when compared to poultry and crops [13,47,48]. On another note, extreme seasonality forces agricultural farmers to sell their products in less demanded markets due to a lack of storage facilities and technologies [[49], [50], [51]]. Conversely, aquaculture boasts a more consistent market and product price compared to livestock and agriculture. While agricultural and livestock products are marketed by syndicate systems [52,53], open marketing is adopted for perishable fish depending on demand and supply, which brings better benefits for farmers [48]. Moreover, fish farmers are getting a better market price from the recently evolved live fish transportation system (with aeration) that allows conveying fish directly from farms to city markets, avoiding the influence of middlemen who previously influenced the aquaculture supply chain [54,55]. However, while the COVID pandemic reduced the consumption of fish in cities, the demand for meat, milk, eggs, and their value-added products remains high [55,56], and some believe that livestock farming would bring the highest profit and reduce poverty at its best.

### 3.4 Policy recommendations from community people

In this quantitative benchmark comparison study, the results showed that the aquaculture sector outperforms the agriculture and livestock sectors significantly in all the considered factors. Therefore, we solely took aquaculture into consideration while capturing information on the needs and institutional supports to help the government in bringing post-COVID livelihood resilience back and allowing access to existing and requested resources.

Through the interviews, the study presents 28 policy recommendations under four broad umbrellas: financial support, technical support, marketing and logistic support, and governance and institutional support (Table 4). Within the existing capital components and resources, respondents identified the provision of bank loans with no/low interest, an aquaculture electricity billing scheme (like agricultural terms), mobile fish clinics for disease diagnosis and treatment, and digital marketing systems as of the highest priority. In the next tier, respondents demanded several supports for devising root-oriented policies, such as a more centralized banking system with easy access to banking services, financial subsidies in case of disasters, training for practicing good aquaculture practices, standards and certifications schemes for occupying international markets, and a traceability scheme to maintain the transparency of inputs used and outputs produced for achieving consumers' trust. While stimulus packages, extension services, and infrastructural capacity building had lower basins of attraction in accomplishing sustainable livelihoods, most of the legal frameworks were considered weak tools to gain benefits. However, assembling and implementing the biosecurity act for aquaculture and regulations on good aquaculture practices and best manufacturing practices in national policies/action plans were ranked as average in improving socio-economy and livelihood resilience.

**Table 4:** Policy recommendations made by aquaculture stakeholders with their average recommendation values on a 5-point liker scale.

Categories	Strategies	Average recommendation values (%)^∗^
Financial support		
F1	Easy access to bank loan	3.97 ± 0.35
F2	Life insurance and emergency funds	2.91 ± 0.19
F3	Provision of low/no interest loan	4.88 ± 0.16
F4	Stimulus package for newcomers	2.90 ± 0.88
F5	Incentives and subsidies in mishap	4.13 ± 0.54
F6	Waiving tax on aquaculture business	2.80 ± 0.98
F7	Billing farm electricity under agricultural terms	4.76 ± 0.22
**Technical support**		
T1	Training facilities	4.36 ± 0.62
T2	Online-based suggestion centre	3.12 ± 0.93
T3	Mobile fish clinic and disease diagnosis	4.66 ± 0.24
T4	Knowledge production and extension services	2.36 ± 1.08
T5	Model farms and demonstration	2.89 ± 1.01
**Marketing and logistic support**		
ML1	Tighter market controlling and monitoring	3.46 ± 0.67
ML2	Strengthening international market	3.91 ± 0.92
ML3	Online markets and home services	4.56 ± 0.41
ML4	Value addition and product development	3.87 ± 1.21
ML5	Infrastructural development and capacity development	2.13 ± 0.98
ML6	Traceability and transparency of inputs and outputs	3.87 ± 1.09
ML7	Contactless fish marketing	3.76 ± 1.03
ML8	Acccredited processing plant development in the localities	2.56 ± 0.98
**Governance and institutional support**		
GI1	Policy and guidelines for GAP and BAP	3.13 ± 1.07
GI2	Standards development and certification	3.53 ± 1.21
GI3	Drug and feed use policy	3.98 ± 0.88
GI4	Water allocation policy	1.11 ± 0.96
GI5	Amendment of existing rules and regulations	1.78 ± 0.34
GI6	Strengthening network among stakeholders and transdisciplinary dialogues	1.49 ± 0.33
GI7	Awareness and healthcare facilities for farm workers	1.32 ± 0.22
GI8	Biosecurity act	2.96 ± 1.13

^∗^100*×* (Likert value × number of individual suggested)/total recommendation value.

In the aquafarming sector, there is a gap between aquaculture and policy framing at individual and community levels, known as the people-policy gap [57]. Therefore, the government must introduce supportive policies to leverage the potential of aquaculture in promoting safe and resilient food systems and livelihoods. The most significant constraint faced by aquaculture farmers, as frequently highlighted in literature, is the financial challenge. To address this, the government of Bangladesh recently announced a long-term loan scheme (at only 4 % interest) for farmers and processors. However, the complex disbursement terms and time-consuming processing limit the scope for aquaculture farmers. Therefore, the government should pass an ordinance to the central bank to issue a specialized loan for fish farmers with a focus on a one-stop service. Most of the subsidies sanctioned by the government, including the COVID subsidy, have failed due to political influences and mismanagement [58]. The government must avoid trading off farmers' loan advantages for political growth and introduce a recuperation fund policy for disaster management, such as the ELAP fund of the USA or the Relief fund of China [59].

Similar to the Agricultural Information and Communication Centre, an ICT-based information center should be developed to facilitate remote services for aquaculture farmers. To weaken syndicate control over market price, policies regarding well-shared benefits should receive sufficient attention. Many scientists have argued for an equitable benefit-sharing policy, but a fair benefit scheme would be more appropriate to ensure the maximum profit share for marginalized farmers.

## 4 Conclusion

The global impact of the COVID-19 pandemic has inflicted significant hardship on livelihoods, particularly in low- and middle-income countries like Bangladesh, where poor recovery rates exacerbate the disproportionate effects. The migration of individuals to rural areas during the pandemic has been hampered by factors such as inadequate preparedness, lack of institutional support, global political unrest, and limited access to financial resources, contributing to significant challenges in sustaining the livelihoods of many. Therefore, assessing potential job options is crucial to bring livelihood resilience back in the post-COVID era and to inform policy people for institutional support. To explore near-shore sustainable livelihood options, two sequential surveys were conducted to present the impact of COVID on the job market and the subsequent job transitions in the Mymensingh district of Bangladesh. Utilizing the SLA framework, we empirically examined various options that could foster long-term resilience, while also identifying local community needs and existing support systems.

The study reported that the pandemic disproportionately affected vulnerable communities, such as seasonal and migrant workers, those with lower educational qualifications, higher age, and women. Furthermore, certain industries such as brick mills, building, and construction were hit hardest by the pandemic. During the COVID situation, a large portion of migrated people adopted agriculture, aquaculture, livestock farming, and small businesses as their means of livelihood due to optimal support from natural resources and established settings in rural areas. However, a significant number of respondents expressed dissatisfaction with their in-COVID jobs and were likely to change their occupations. Out of the 13 job categories preferred in the post-COVID era, aquaculture, agriculture, and livestock farming remained the most favored, with aquaculture being the top choice.

The study emphasized the importance of preferred occupations in achieving post-COVID livelihood resilience and promoting lives in rural Bangladesh. Aquaculture and livestock farming were considered better options for supporting the five capitals needed to ensure sustainable livelihoods in the post-COVID era. Aquaculture was reported to be more gender-inclusive than other options, offering better benefits for employees. However, rural communities asked for some policy interventions to make their livelihoods more resilient for aquaculture business. Among the 28 policy recommendations provided by community members, priority was given to bank loans, an aquaculture electricity billing scheme, mobile fish clinics, and digital marketing systems. While policies related to biosecurity, standards and certification schemes, and traceability were acknowledged, legal frameworks were considered weak tools.

Despite limitations in covering large geographical areas stemming from COVID-related constraints and a limited timeframe, this study contributes valuable insights into post-pandemic job opportunities and support needs in rural Bangladesh. We urge policymakers to promptly provide institutional and financial support to COVID-affected rural communities, facilitating the restoration of livelihood resilience.

## Data availability

Data will be made available on request.

## CRediT authorship contribution statement

**Abul Bashar:** Writing – review & editing, Writing – original draft, Investigation, Formal analysis, Conceptualization. **Neaz A. Hasan:** Writing – review & editing, Data curation. **Mohammad Mahfujul Haque:** Supervision, Resources, Project administration, Investigation, Funding acquisition.

## Declaration of competing interest

The authors declare that they have no known competing financial interests or personal relationships that could have appeared to influence the work reported in this paper.
