# Supplementary material for: Exploring sustainable livelihood options for COVID-impacted rural communities in Bangladesh
Source: Heliyon. 2024 Sep 27;10(19):e38664. doi: 10.1016/j.heliyon.2024.e38664 (PMC11471468; doi:10.1016/j.heliyon.2024.e38664)
Supplement: Multimedia component 1 [file mmc1.docx]

This will consist of face-to-face/telephone interviews of approx 250 people. We will explain what the project is about and why their input is so important and then record the information upon signing the consent form.

| Survey code: | BAU/COVID_LIV/__ | | |  |
| --- | --- | --- | --- | --- |
| Interviewer’s name: |  | Interview date: |  | |

**SECTION A: ABOUT YOU AND YOUR FARM**

| 1. Interviewee’s name: |  |
| --- | --- |
| 2. Interviewee’s age: | /Years |
| 3. Interviewee’s gender: | Male Female |
| 4. Level of education: | No education Primary Secondary  Higher secondary University  6=Other: (Specify_________________________________________) |
| 5. Farm address: | Village: Union: Upazila: |
| 6. GPS: | N: o ’ ’’ E: o ’ ’’ |
| 7. Contact details: | Mobile No: |
|  | Email: |
| 8. Size of households |  |

**SECTION B: YOUR OCCUPATION AND TRANSITION**

| 9. Pre-COVID Job: | Position: Job type: Local Migrant |
| --- | --- |
|  | Employers Name and Address: |
|  | No. of years engaged: Last salary (BDT): |
| 10. Are you still engaged in Pre-COVID job: Yes No | |
| 11. In-COVID Job: | Position: Job type: Local Migrant |
|  | Employers Name and Address: |
|  | Current salary (BDT): |
| 12. Are you satisfied with your job? Yes No. | |
| 13. If not, towards which profession would you like to move? Mention the reason(s) behind. | |
