# Supplementary material for: Exploring sustainable livelihood options for COVID-impacted rural communities in Bangladesh
Source: Heliyon. 2024 Sep 27;10(19):e38664. doi: 10.1016/j.heliyon.2024.e38664 (PMC11471468; doi:10.1016/j.heliyon.2024.e38664)
Supplement: Multimedia component 2 [file mmc2.docx]

**Introduction**

Introduce the research project and our overall project objectives:

- what are we seeking to achieve?
- the importance of participant involvement
- what we hope the project will help to deliver

| Survey code: | BAU/COVID_LIV/__ | | |  |
| --- | --- | --- | --- | --- |
| Interviewer’s name: |  | Interview date: |  | |

Explain the concept of SLA and show them all identified livelihood components under five capitals. Ask them if they can identify anything else and then to assign individual weight on a 5-point scale (1 for very poor, 2 for poor, 3 for moderate, 4 for strong, and 5 for very strong) to show their degree of impacts on their livelihood.

Respondents will be asked to provide indicator value of each component needed for earning sustainable livelihood outcomes from agriculture, aquaculture and livestock farming.

| Capitals | Components | Assigned score | Indicator value for | | |
| --- | --- | --- | --- | --- | --- |
|  |  |  | Agriculture | Aquaculture | Livestock farming |
| Physical | Basic infrastructure for farming |  |  |  |  |
|  | Transportation and logistics support |  |  |  |  |
|  | Input supply and availability |  |  |  |  |
|  | Market stability and access |  |  |  |  |
|  | Electricity and power supply |  |  |  |  |
|  | Value addition facilities |  |  |  |  |
|  | Others (if any) |  |  |  |  |
| Natural | Availability of arable lands |  |  |  |  |
|  | Meteorological and climatic conditions |  |  |  |  |
|  | Water availability and supply |  |  |  |  |
|  | Flood control and storm protection |  |  |  |  |
|  | Ecosystem services |  |  |  |  |
|  | Disease history and management |  |  |  |  |
|  | Resource management |  |  |  |  |
|  | Others (if any) |  |  |  |  |
| Financial | Capital support (bank loan, easy access to credits) |  |  |  |  |
|  | Economic return (BCR) |  |  |  |  |
|  | National subsidies |  |  |  |  |
|  | Alternative income generation opportunity |  |  |  |  |
|  | Insurance |  |  |  |  |
|  | Others (if any) |  |  |  |  |
| Human | Education, skill, and knowledge |  |  |  |  |
|  | Physical compatibility |  |  |  |  |
|  | Ability to work/labor availability |  |  |  |  |
|  | Mental and physical health support |  |  |  |  |
|  | Others (if any) |  |  |  |  |
| Social | Collaboration, networking, and cooperation |  |  |  |  |
|  | Social security |  |  |  |  |
|  | Gender issues |  |  |  |  |
|  | Social hierarchy and evaluation |  |  |  |  |
|  | Others (if any) |  |  |  |  |
