# Supplementary material for: Exploring sustainable livelihood options for COVID-impacted rural communities in Bangladesh
Source: Heliyon. 2024 Sep 27;10(19):e38664. doi: 10.1016/j.heliyon.2024.e38664 (PMC11471468; doi:10.1016/j.heliyon.2024.e38664)
Supplement: Multimedia component 3 [file mmc3.docx]

**Introduction**

Introduce the research project and our overall project objectives:

- what are we seeking to achieve?
- the importance of participant involvement
- what we hope the project will help to deliver

| Survey code: | BAU/COVID_LIV/__ | | |  |
| --- | --- | --- | --- | --- |
| Interviewer’s name: |  | Interview date: |  | |

Explain the concept of policy recommendation, ask them to identify potential interventions/amendments needed for sustainable livelihood outcomes and to assign relative importance for each intervention.

| Interventions | Relative importance (1-5) |
| --- | --- |
|  |  |
| 1. |  |
| 2. |  |
| 3. |  |
